# Gastro-splenic fistula, a rare presentation of sickle cell disease patient with splenic abscess: Case report and literature review

**DOI:** 10.1016/j.ijscr.2019.02.039

**Published:** 2019-03-05

**Authors:** Ali Alsinan, Moslem Alelq, Fozia Alsugair, Ali Hassan, Abrar Waheed, Saeed Alshomimi

**Affiliations:** Department of Surgery, King Fahd Hospital of the University, Imam Abdulrahman Bin Faisal University, Saudi Arabia

**Keywords:** Fistula, Sickle cell disease, Splenectomy, Endoscopy

## Abstract

**Introduction:**

Hemoglobinopathies such as Thalassemia and Sickle Cell Disease are risk factors for splenic abscess formation. Gastro-splenic fistula is a rare complication of splenic abscess. In the literature, there are no reported cases of gastro-splenic fistula in patients with hemoglobinopathies.

**Presentation of case:**

We are presenting a rare case of a 15-year-old boy with undiagnosed sickle cell trait with splenic sequestration crisis as first presentation of his disease. This was complicated by splenic abscess formation. Gastro-Splenic fistula was not apparent in computed tomography and upper gastrointestinal endoscopy findings were misinterpreted initially, which lead to delay in diagnosis and management. The patient was managed initially with antibiotics, followed by splenectomy and partial gastrectomy.

**Discussion:**

Gastro-splenic fistula is not a common entity. It may be caused by gastric or splenic pathologies. In our case, which is the first reported in literature, the fistula developed as a complication of splenic abscess in patient with sickle cell disease. Its presentation depends on the underlying condition. CT scan is the modality of choice for diagnosis. Upper GI endoscopy findings of the fistula is often misinterpreted. The treatment of fistula is splenectomy and partial gastrectomy.

**Conclusion:**

Delayed diagnosis and management of splenic abscess in patients with sickle cell disease predisposes to fistula formation.

## Highlights

• Gastro-splenic fistula is rare condition that develops as complication of gastric or splenic pathologies.
• Delayed diagnosis of splenic abscess predisposes to the formation of gastro-splenic fistula.
• Gastro-splenic fistula could develop in patients with sickle cell disease.
• CT scan is the diagnostic modality of choice and upper GI endoscopy findings are often misinterpreted.
• Splenectomy and proximal gastrectomy is the treatment of choice for the gastro-splenic fistula.

## 1 Introduction

Hemoglobinopathies such as thalassemia and sickle cell disease are prevalent in the eastern region of Saudi Arabia with sickle cell trait accounting for up to 17% of the population [1]. Herein, we present a very rare complication of sickle cell trait in which a patient developed a splenic abscess eroding the stomach. This work has been reported in line with the SCARE criteria [2].

## 2 Presentation of case

A previously well 15-year-old boy presented with sudden onset of left-sided abdominal pain and symptoms of anemia. His physical examination was remarkable for massive splenomegaly and his laboratory investigations were significant for low hemoglobin level (7 g/dl). Hemoglobin S level of 45% and Hemoglobin F level of 15% by HPLC (High-Performance Liquid Chromatography) confirming the diagnosis of Sickle Cell Trait. The patient denied any previous complaints or hospital admissions related to sickle cell disease. He was admitted to the Intensive Care Unit (ICU) in another hospital where he received blood transfusion and referred to our hospital for further management. Three weeks later upon presentation to our hospital, he was febrile, pale and jaundiced. Abdominal examination revealed left hypochondriac tenderness and massive splenomegaly.

**Fig. 1:**
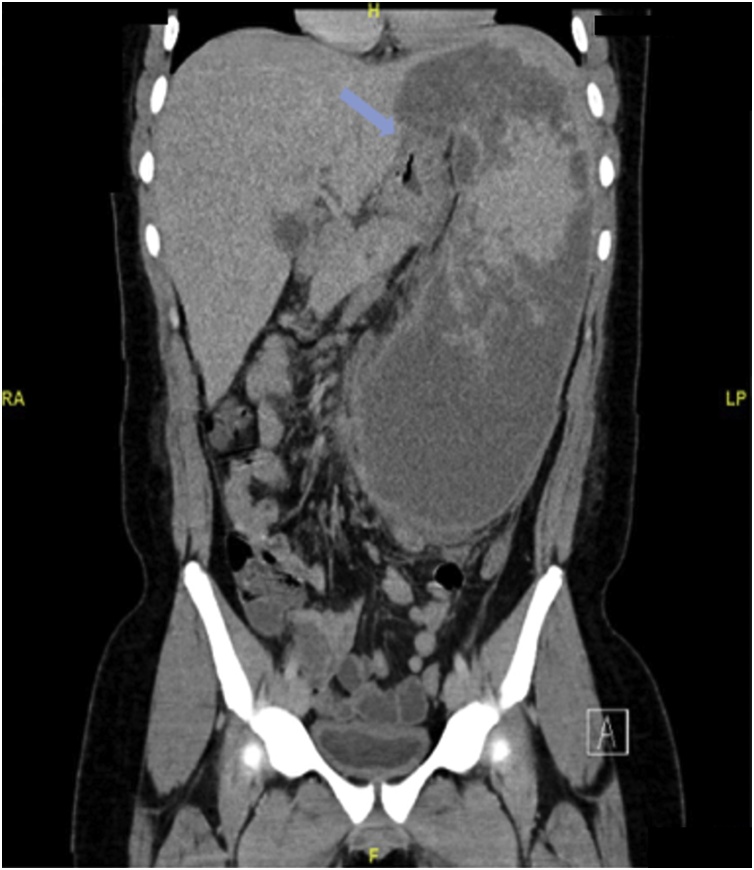
Coronal view of abdominal CT scan with IV contrast showing massive splenomegaly with query indentation of upper part of the stomach.

**Fig. 2:**
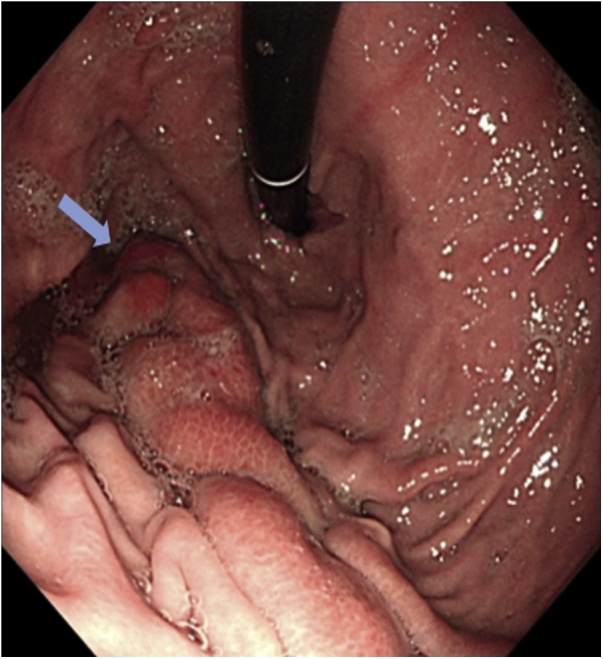
Endoscopic picture of the stomach showing edematous folds with query fistula opening in the funds area.

**Fig. 3:**
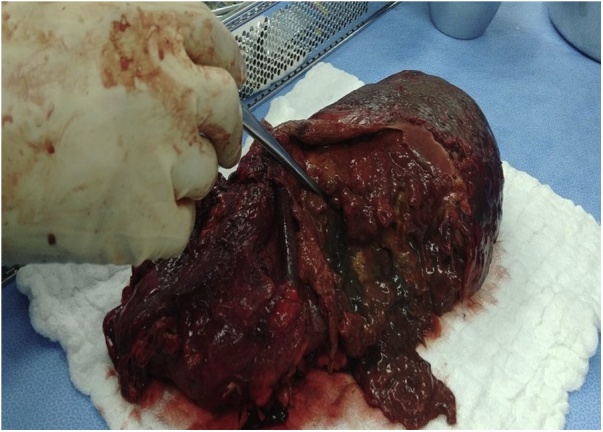
Gross picture of the spleen after removal, showing huge abscess cavity.

**Fig. 4:**
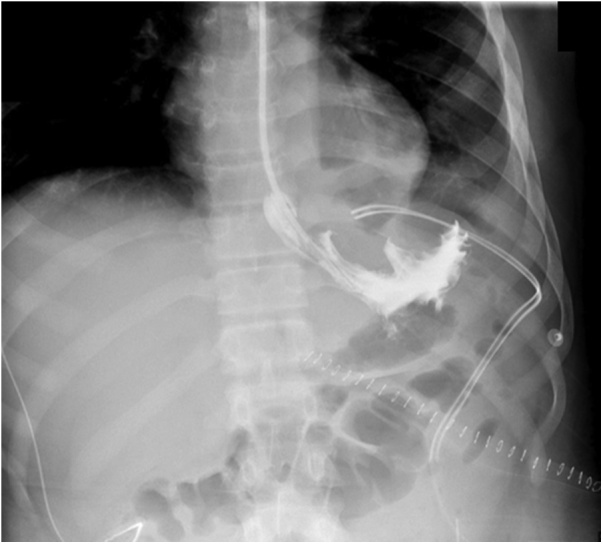
Contrast meal study demonstrates no leak in the gastric surgical site.

A Computed Tomography (CT) Scan of the abdomen showed massive splenomegaly with splenic infarction and possible superimposed infection (Fig. 1). The plan for further management was taken after the discussion in a multi-disciplinary team consisting of hematologist, surgeons, gastroenterologist, intensivist and infectious disease consultant. The patient was started on hydration, proper analgesia and antibiotics with a plan for operation after his condition improves. Prior to surgery, he had an episode of vomiting which was thought to be coffee-ground vomitus; accordingly, upper GI endoscopy was done and the edematous gastric mucosa in the fundus was misinterpreted as large gastric varices at the time of the study (Fig. 2). Patient underwent open splenectomy under general anesthesia after being prepared with hydration, analgesia, simple blood transfusion and incentive spirometry to prevent perioperative complications. During the operation, multiple infarcts and abscesses were seen. The spleen was fused to the stomach. Upon dissection of the adhesions, a fistulous tract was found connecting the stomach fundus to the spleen with copious amount of pus coming out. The fistula was managed by partial gastrectomy (Fig. 3). Three days post-operative, contrast meal study was done which demonstrated no leak at the surgical site (Fig. 4).

The patient had a smooth recovery period and was discharged home 10 days post-operatively.

## 3 Discussion

Patients with hemoglobinopathies, particularly sickle cell disease usually present with mild splenomegaly during the first decade of life, which then undergoes progressive atrophy leading to autosplenectomy due to repeated attacks of vaso-occlusion and infarction. However, splenic complications in patients with sickle cell disease are not very uncommon and patients can present with varying degrees of splenomegaly. These include a spectrum of disorders such as splenic infarction, hypersplenism, acute splenic sequestration crisis and splenic abscess, which can be complicated by fistula formation [3].

Splenic abscess per se in patients with sickle cell disease is considered to be very rare with prevalence around 0.7% in autopsy studies [4]. The diagnosis can be delayed or missed in these patients as the presentation can be initially attributed to vaso-occlusive crisis and splenic infarction.

Gastro-splenic fistula is an uncommon entity and is usually caused by many underlying conditions. The pathogenic mechanism involves progressive infiltration, erosion and penetration of the stomach wall. Because of the close proximity between the gastric fundus and the spleen a fistulous tract can be formed [5]. This can occur with either gastric or splenic pathologies.

For gastric pathologies, this mechanism can explain the reported cases of gastro-splenic fistula caused by malignant conditions such as lymphoma and gastric adenocarcinoma [6,7], in addition to benign conditions such as gastric ulcers and Crohn's disease [5].

For splenic pathologies, causes include splenic lymphoma and splenic abscess, which can be predisposed by haemoglobinopathies such as sickle cell disease/trait, trauma or sepsis.

In cases of gastro-splenic fistula, usually the presentation is of the underlying condition, not for the fistula itself. Patients usually present with abdominal pain, tenderness and fever [8]. Splenomegaly is only found in half of the cases [9]. Upper GI bleeding is a rare presentation but can lead to serious consequences [10].

CT scan is the modality of choice for the diagnosis of gastro-splenic fistula. Upper GI endoscopy can visualize the fistula opening [11], but because of the rarity of the condition and because of edematous mucosa, findings might be misinterpreted as varices or ulcers (as in our case). Ultrasound can only visualize splenic collections.

Upon review of literature, we found only three cases of gastro-splenic fistula due to splenic abscess, none of them with hematological disease [5,11,12]. In the largest series of splenectomies in sickle cell disease patients in our area, only 7 out of 173 seen with splenic abscess and needed surgery but none of them had gastric fistula [13].

The mainstream management of gastro-splenic fistula is surgery, in form of splenectomy and partial gastrectomy as in our case. Other forms of management include stapling the involved part of the stomach or dividing the fistula tract and over sewing the stomach [11]. Laparoscopic approach can be utilized, but in cases of massive splenomegaly, open approach is preferred. There are reported cases of conservative management in the form of abscess aspiration but it is advisable to go with surgical management to avoid massive or recurrence of GI bleeding [5,14].

In our patient, we are reporting the first gastro-splenic fistula in splenic abscess occurring after sequestration crisis in a sickle cell trait patient as first presentation of his disease. We believe that our patient could be the first to be reported describing gastro-splenic fistula in a sickle cell trait with splenic abscess due to sequestration crisis as first presentation of the disease.

## 4 Conclusion

Haemoglobinopathies might be a risk factor for splenic abscess formation, and we believe that sequestration crisis and splenic infarction superimposed with infection may lead to it. If the management is delayed, erosion to the gastric wall and a formation of a fistula tract between the spleen and the stomach may occur. This will necessitate a more complex surgical management.

## Conflicts of interest

Nothing to disclose.

## Funding

No source of funding.

## Ethical approval

We had Institutional Review Board approval. Case Report is presented anonymously.

## Consent

Written consent was taken from the patient’s mother (as the patient is minor).

## Author contribution

**Ali Alsinan**: writing the paper.

**Moslem Alelq**: literature review.

**Fozia Alsugair**: editing the paper.

**Ali Hassan**: editing the paper.

**Abrar Waheed**: revision of the paper.

**Saeed Alshomimi**: writing the paper; treating physician of the patient.

## Registration of research studies

Not Applicable (Case Report; not an interventional study).

## Guarantor

Dr. Saeed AlShomimi.

Consultant Gastric Tumours and Upper GI Surgeon.

King Fahd University Hospital, Khobar, Saudi Arabia.

## Provenance and peer review

Not commissioned, externally peer reviewed.
